# High fat diet modifies the association of lipoprotein lipase gene polymorphism with high density lipoprotein cholesterol in an Asian Indian population

**DOI:** 10.1186/s12986-016-0155-1

**Published:** 2017-01-19

**Authors:** K. A. Ayyappa, I. Shatwan, D. Bodhini, L. R. Bramwell, K. Ramya, V. Sudha, R. M. Anjana, J. A. Lovegrove, V. Mohan, V. Radha, K. S. Vimaleswaran

**Affiliations:** 1 Department of Molecular Genetics, Madras Diabetes Research Foundation, Kallam Anji Reddy Centre, Plot no. 20, Golden Jubilee Biotech Park for Women Society, SIPCOT-IT PARK, Siruseri, Chennai 603 103 India; 2 Department of Food and Nutritional Sciences, Hugh Sinclair Unit of Human Nutrition and Institute for Cardiovascular and Metabolic Research (ICMR), University of Reading, Whiteknights, PO Box 226, Reading, RG6 6AP UK; 3 Food and Nutrition Department, Faculty of Home Economics, King Abdulaziz University, Jeddah, Saudi Arabia; 4 Department of Foods, Nutrition and Dietetics Research, Madras Diabetes Research Foundation, Chennai, India; 5 Department of Diabetology, Madras Diabetes Research Foundation, Chennai, India; 6 Dr. Mohan’s Diabetes Specialties Centre, WHO Collaborating Centre for Non-communicable Diseases Prevention and Control, Chennai, India; 7 Present Address: Department of Biotechnology, School of Chemical & Biotechnology, SASTRA University, Tanjore, India

**Keywords:** *LPL*, Lipoprotein lipase, Asian Indians, HDL-C, Triacylglycerol, CURES, Lipids, Dietary intake

## Abstract

**Background:**

Single nucleotide polymorphisms (SNPs) in lipoprotein lipase gene (*LPL*) have been shown to influence metabolism related to lipid phenotypes. Dietary factors have been shown to modify the association between *LPL* SNPs and lipids; however, to date, there are no studies in South Asians. Hence, we tested for the association of four common *LPL* SNPs with plasma lipids and examined the interactions between the SNPs and dietary factors on lipids in 1,845 Asian Indians.

**Methods:**

The analysis was performed in 788 Type 2 diabetes cases and 1,057 controls randomly chosen from the cross-sectional Chennai Urban Rural Epidemiological Study. Serum triacylglycerol (TAG), serum total cholesterol, and high-density lipoprotein cholesterol (HDL-C) were measured using a Hitachi-912 autoanalyzer (Roche Diagnostics GmbH, Mannheim, Germany). Dietary intake was assessed using a semi-quantitative food frequency questionnaire. The SNPs (rs1121923, rs328, rs4922115 and rs285) were genotyped by polymerase chain reaction followed by restriction enzyme digestion and 20% of samples were sequenced to validate the genotypes obtained. Statistical Package for Social Sciences for Windows version 22.0 (SPSS, Chicago, IL) was used for statistical analysis.

**Results:**

After correction for multiple testing and adjusting for potential confounders, SNPs rs328 and rs285 showed association with HDL-C (*P* = 0.0004) and serum TAG (*P* = 1×10^−5^), respectively. The interaction between SNP rs1121923 and fat intake (energy %) on HDL-C (*P* = 0.003) was also significant, where, among those who consumed a high fat diet (28.4 ± 2.5%), the T allele carriers (TT + XT) had significantly higher HDL-C concentrations (*P* = 0.0002) and 30% reduced risk of low HDL-C levels compared to the CC homozygotes. None of the interactions on other lipid traits were statistically significant.

**Conclusion:**

Our findings suggest that individuals carrying T allele of the SNP rs1121923 have increased HDL-C levels when consuming a high fat diet compared to CC homozygotes. Our finding warrants confirmation in prospective studies and randomized controlled trials.

**Electronic supplementary material:**

The online version of this article (doi:10.1186/s12986-016-0155-1) contains supplementary material, which is available to authorized users.

## Background

The Asian Indian population has a greater predisposition to non-communicable diseases such as type 2 diabetes (T2D) [1, 2] and cardiovascular disease (CVD) [3] compared to Europeans. Despite low body mass index (BMI), Indians are characterized by a higher frequency of hyperinsulinemia [4], insulin resistance [5], dyslipidemia with hypertriacylglycerolemia and low high-density lipoprotein cholesterol (HDL-C) levels [6] and increased visceral fat, which are referred to as ‘Asian Indian Phenotype’ or ‘Atherogenic Lipoprotein Phenotype’ [7, 8]. Blood lipid levels are heritable phenotypes and findings from previous studies show that the blood concentrations of HDL-C, low density lipoprotein cholesterol (LDL-C) and triacylglycerol (TAG) have a strong inheritance [9].

Genetic studies have implicated several gene loci in the predisposition to dyslipidemia in Asian Indians [10–13], one of which is lipoprotein lipase (*LPL*) [14–16]. LPL plays an important role in the metabolism of HDL-C, where it has been shown to hydrolyze TAG in TAG-rich lipoproteins such as chylomicrons and very low density lipoproteins [17]. It has been postulated that increased activity of LPL enzyme enhances the release of components of TAG-rich lipoproteins which are then transferred to HDL to raise HDL levels; conversely, lack of LPL can retard the transfer of these components to HDL [18]. Several candidate gene studies have shown an association between single nucleotide polymorphisms (SNPs) in *LPL* and lipid traits in various populations including Asian Indians [11, 12, 19–22]. Genome wide association studies have also demonstrated strong evidence for the association of *LPL* polymorphisms with HDL-C concentrations [23–25]. A few studies have examined the *LPL* gene–diet interactions in association with HDL-C [26–30]; however, the findings have been quite inconsistent due to variations in sample size, dietary factors and the selection of *LPL* polymorphisms.

Given that there are no gene-diet interaction studies, to date, in Asian Indian populations, we examined the association of four common *LPL* SNPs [Val135Val C/T (rs1121923), Ser447Ter C/G (rs328), G/A (rs4922115) and *Pvu II* C/T (rs285)] with HDL-C and investigated the interactions of these four polymorphisms with dietary carbohydrate, fat and protein percentage on HDL-C in up to 1,845 participants (788 T2D cases and 1,057 controls) from the cross-sectional Chennai Urban Rural Epidemiological Study (CURES). In addition, we examined the genetic associations and interactions for other lipid traits such as TAG, LDL-C and total cholesterol in these participants.

## Methods

### Study population

One thousand eight hundred and forty five participants comprising 788 cases with T2D and 1,057 controls with normal glucose tolerance (NGT) were randomly chosen from the urban component of the Chennai Urban Rural Epidemiological Study (CURES), an epidemiological study conducted on a representative population (age >20 years) of Chennai (formerly Madras), the fourth largest city in India. The detailed methodology of the study participants is published elsewhere [31]. Briefly, in Phase 1 of CURES, 26,001 individuals were recruited based on a systematic random sampling technique. Participants with self-reported diabetes taking drug treatment for diabetes were classified as “known diabetes subjects.” All known diabetes participants (*n* = 1,529) were invited to visit the center for detailed studies. In addition, every 10th individual of the 26,001 individuals without known diabetes was invited to undergo oral glucose tolerance tests using a 75-g oral glucose load (dissolved in 250 ml of water) (Phase 3 of CURES). Those who were confirmed by oral glucose tolerance test to have 2-h plasma glucose value ≥11.1 mmol/l based on World Health Organization (WHO) consulting group criteria were labeled as “newly detected diabetes subjects” and those with 2-h plasma glucose value <7.8 mmol/l as being NGT [32]. CURES participants who were on lipid lowering drugs such as statins, fibrates and niacin were excluded from the study (*n* = 134). On the basis of the National Cholesterol Education Program-Adult Treatment Panel III (NCEP-ATP III) guidelines [33] the study population was divided into those with normal HDL-C (≥1.03 mmol/l for men; ≥1.3 mmol/l for women) and low HDL-C (<1.03 mmol/l for men; <1.3 mmol/l for women). Written informed consent was obtained from each study participant, and the study was approved by the Madras Diabetes Research Foundation Institutional Ethics Committee.

### Phenotype measurements

Anthropometric measurements including weight, height, and waist were obtained using standardized techniques. The BMI was calculated as weight (in kg) divided by the square of height (in m). Biochemical analyses were performed on a Hitachi-912 Auto Analyzer (Hitachi, Mannheim, Germany) using kits supplied by Roche Diagnostics (Mannheim). Fasting plasma glucose (glucose oxidase–peroxidase method), serum total cholesterol (cholesterol oxidase-phenol-4-amino-antipyrene peroxidase method), serum TAG (glycerol phosphatase oxidase-phenol-4-amino-antipyrene peroxidase method), and HDL-C (direct method; polyethylene glycol-pretreated enzymes) were measured. Low-density lipoprotein cholesterol was calculated using the Friedewald formula [34]. Glycated haemoglobin (HbA1c) was estimated by high-performance liquid chromatography using a Variant™ machine (Bio-Rad, Hercules, CA, USA). Serum insulin concentration was estimated using an enzyme-linked immunosorbent assay (Dako, Glostrup, Denmark).

### Dietary assessment

Dietary intakes were assessed using a previously validated and published [35] interviewer administered semi-quantitative food frequency questionnaire (FFQ) containing 222 food items to estimate food intake over the past year. Briefly, individuals were asked to estimate the usual frequency (number of times per day, week, month or year**/**never) and the usual serving size of the portion of the various food items in the FFQ. Common household measures such as household cups, bowls, ladles, spoons (for the cooked foods like vegetables), wedges, circles of different diameter and visual atlas of different sizes of fruits (small, medium, large) were shown to assist the individuals in estimating portions. A detailed description of the development of FFQ and the data on reproducibility and validity had been published [35]. EpiNu, an in-house database was used to assess the average daily food and nutrient intake.

### SNP selection and genotyping

Four common SNPs in the *LPL* gene (rs285, rs328, rs4922115 and rs1121923) were chosen for the present study. The SNPs rs328 and rs285 were chosen based on their previous associations with lipid outcomes in several populations [11, 12, 22, 36]. The SNPs rs1121923 and rs4922115 were identified from the dbSNP database (http://www.ncbi.nlm.nih.gov/SNP/) based on their location in the exon 3 and 3’UTR regions, respectively, assuming that variations in the coding and regulatory regions might confer a functional effect on the gene expression. The SNPs were genotyped by polymerase chain reaction on a GeneAmp® PCR system 9700 thermal cycler (Applied Biosystems, Foster City, CA) followed by restriction enzyme digestion (New England Biolabs, Inc., Beverly, MA). The program usually had the following steps: initial denaturation at 95 °C for 10 min, 30–35 cycles of denaturation at 95 °C for 45 s, primer-annealing at 58 °C for rs1121923 and rs285 SNPs and 60 °C for rs328 and rs4922115 SNPs for 45 s, and primer extension at 72 °C for 45 s, followed by a final extension at 72 °C for 5 min. The restrictions enzymes used for genotyping the SNPs were *Sau96I* for rs1121923, *Mnl I* enzyme for rs328, the *Eco RV* enzyme for rs4922115 and *Pvu II* for rs285. Agarose gel electrophoresis was used to detect the amplification of PCR reaction and the restriction enzyme digested products. To ensure that the genotyping was of adequate quality, we performed random duplicates in 10% of the samples. The assays were performed by a technician who was masked to the phenotype, and there was 98% concordance in the genotyping. Variants were also confirmed by direct sequencing using an ABI 3500 genetic analyzer (Applied Biosystems, Foster City, CA). Population stratification was performed using a case–control approach at 6 unlinked marker loci believed to be unrelated to the disease under study, but known to have allelic diversity among different populations [37].

### Statistical analysis

Statistical Package for Social Sciences for Windows version 22.0 (SPSS, Chicago, IL) was used for statistical analysis. The effects of the variants on quantitative and categorical variables were analyzed. Allele frequencies were estimated by gene counting. Agreement with Hardy–Weinberg equilibrium (HWE) expectations was tested using a χ2goodness-of-fit test. Comparison of the means between the two groups was analyzed by independent *t-*test. The *χ*2 test was used to compare the proportions of genotypes or alleles. Dominant model was used, given the low frequency of minor allele homozygotes. Linear regression was used to examine the association of the *LPL* SNPs with various lipid outcomes. The SNP-diet interactions on lipid traits were tested by including the interaction term in linear regression models. Models were adjusted for age, gender, BMI (as continuous), T2D status and total energy intake wherever appropriate. Multiple testing correction using Bonferroni method was applied separately for the testing of main and interaction effects (i.e., association of the four SNPs with HDL-C and interaction with dietary factors on HDL-C levels) [*P* ≤ 0.003 (=0.05/20) was considered statistically significant] and additional analyses (i.e., association of the four SNPs with other lipid traits and interaction with dietary factors on other lipid traits) [*P* ≤ 0.001 (=0.05/48) was considered statistically significant].

## Results

Table 1 shows the anthropometric and biochemical characteristics of NGT and T2D participants. T2D cases had markedly increased levels of TAG, LDL-C and total cholesterol, while HDL-C was significantly lower in cases compared to controls (*P* < 0.003 for all comparisons). In our study population, the genotype distributions for the four *LPL* polymorphisms were GG: 68.5%, GA: 27.5% and AA: 4.0% (rs4922115); CC: 87.5%, CT: 12.0% and TT: 0.5% (rs1121923); CC: 72.8%, CG: 25.2% and GG: 2.00% (rs328); and CC: 40.9%, CT: 45.6% and TT: 13.5% (rs285). All the four SNPs were in HWE (*P* > 0.05).

**Table 1:** Baseline characteristics of the CURES study participants

	Participants with normal glucose tolerance (*N* = 1,057)	Participants with type 2 diabetes (*N* = 788)	*P* value
Age (year)	38.5 ± 13.6	50.6 ± 11.1	<0.0001
Gender (men/women)	608/ 449	433/355	0.2*
BMI (kg/m^2^)	23.2 ± 4.5	25.2 ± 4.4	<0.0001
Fasting Glucose (mmol/l)	4.7 ± 0.5	8.8 ± 3.8	<0.0001
Fasting Insulin (lIU/mL)	8.2 ± 5.6	11.6 ± 7.0	<0.0001
Total serum Cholesterol (mmol/l)	4.6 ± 0.9	5.2 ± 1.1	<0.0001
Serum TAG (mmol/l)	1.3 ± 0.7	1.9 ± 1.3	<0.0001
HDL-C (mmol/l)	1.12 ± 0.25	1.08 ± 0.24	0.003
LDL-C (mmol/l)	2.9 ± 0.8	3.2 ± 0.9	<0.0001
Glycated hemoglobin (%)	5.56 ± 0.47	8.64 ± 2.26	<0.0001
Systolic pressure (mmHg)	117.0 ± 17.4	128.9 ± 21.5	<0.0001
Diastolic pressure (mmHg)	73.2 ± 11.2	77.1 ± 12.0	<0.0001
Protein intake (energy %)	11.3 ± 1.2	11.4 ± 1.2	0.03
Carbohydrate intake (energy %)	64.4 ± 6.4	64.9 ± 5.8	0.1
Fat intake (energy %)	23.5 ± 4.7	23.4 ± 4.6	0.8
Total energy intake (kcal)	2627.2 ± 725.4	2533.5 ± 907.2	0.02
Total saturated fat intake (%)	2.4 ± 0.9	2.1 ± 0.9	<0.0001
Total monounsaturated fat (%)	1.9 ± 0.7	1.7 ± 0.8	0.0002
Total polyunsaturated fat (%)	1.6 ± 0.8	1.7 ± 0.9	0.04

Data shown are represented as means ± SD, wherever appropriate

*P* values for the differences in the means/ proportions between cases and controls

*P* values were calculated by using Independent *t* test

*P value was calculated using a Chi-square test

*CURES* Chennai Urban Rural Epidemiological Study, *TAG* triacylglycerol, *HDL-C* high density lipoprotein, *LDL-C* low density lipoprotein cholesterol

The association between *LPL* SNPs and HDL-C levels is presented in Table 2. Of the four *LPL* variants, the SNP rs328 alone showed a significant and a consistent association with HDL-C concentrations [both as continuous and categorical variable (stratified based on NCEP ATP III guidelines)] under a dominant model after correction for multiple testing (*P* = 0.0004 for the continuous variable and *P* = 0.001 for the categorical variable). The minor allele (G) carriers of the SNP rs328 had 5% higher HDL-C compared to the homozygous carriers of the common ‘C’ allele.

**Table 2:** Association of the lipoprotein lipase single nucleotide polymorphisms (SNPs) with HDL-C levels

Association of the SNPs with HDL-C levels (continuous variable)		
SNP	HDL-C levels (means ± SD)	
SNP rs4922115		
GG	1.1 ± 0.3	
GA	1.1 ± 0.2	
AA	1.1 ± 0.2	
Dominant model (GG vs GA + AA) (*P* value)	0.02	
SNP rs1121923		
CC	1.1 ± 0.3	
CT	1.2 ± 0.3	
TT	1.0 ± 0.2	
Dominant model (CC vs CT + TT) (*P* value)	0.02	
SNP rs328		
CC	1.1 ± 0.3	
CG	1.2 ± 0.3	
GG	1.2 ± 0.2	
Dominant model (CC vs CG + GG) (*P* value)	**0.0004**	
SNP rs285		
CC	1.1 ± 0.3	
CT	1.1 ± 0.2	
TT	1.2 ± 0.2	
Dominant model (CC vs CT + TT) (*P* value)	0.03	
Association of the SNPs with HDL-C (categorical variable)		
	Low HDL-C levels Number (%)	Normal HDL-C levels Number (%)
SNP rs4922115		
GG	366 (65%)	497 (71.3%)
GA	171 (30.4%)	167 (25.3%)
AA	26 (4.6%)	24 (3.4%)
Dominant model (GG vs GA + AA) (*P* value)	0.02	
SNP rs1121923		
CC	530 (91.7%)	642 (84.7%)
CT	45 (7.8%)	112 (14.8%)
TT	3 (0.5%)	4 (0.5%)
Dominant model (CC vs CT + TT) (*P* value)	0.1	
SNP rs328		
CC	336 (78.3%)	540 (69.8%)
CG	88 (20.5%)	215 (27.8%)
GG	5 (1.2%)	19 (2.5%)
Dominant model (CC vs CG + GG) (P value)	**0.001**	
SNP rs285		
CC	336 (45.6%)	421 (38%)
CT	330 (44.8%)	515 (46.5%)
TT	71 (9.6%)	171 (15.4%)
Dominant model (CC vs CT + TT) (P value)	**0.001**	

*HDL-C* High density lipoprotein cholesterol

*P* values are adjusted for age, gender, body mass index, and Type 2 diabetes status

Those p values that are in bold implicates those values that are significant after Bonferroni correction

**Fig. 1:**
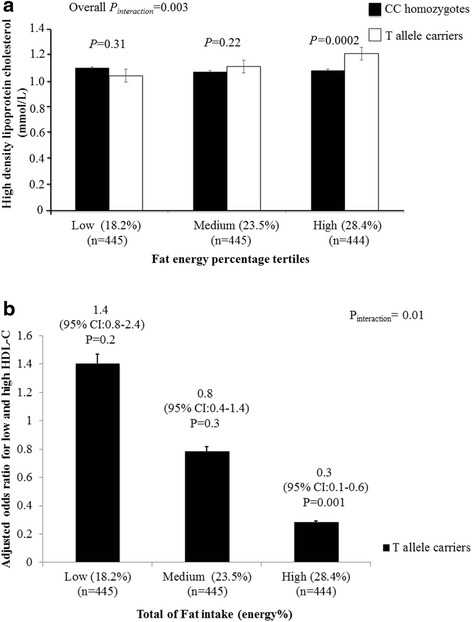
**a** Interaction between Lipoprotein lipase gene SNP rs1121923 and fat energy intake (%) on HDL-C concentrations (P_interaction_ = 0.003). Among those who consumed a high fat diet, T allele carriers had significantly higher levels of HDL-C compared to the CC homozygotes (*P* = 0.0002). **b** Interaction between Lipoprotein lipase gene SNP rs1121923 and fat energy intake (%) on HDL-C as a categorical variable (P_interaction_ = 0.01). Among those who consumed a high fat diet (28.4%), the individuals who carried the T allele have 30% reduced risk of low HDL-C levels compared to those who carry the CC genotype (*P* = 0.001). Data shown are represented as means ± SE. P_interaction_ values adjusted for age, gender, body mass index, type 2 diabetes and total energy intake

In the interaction analysis, after correction for multiple testing, none of the interactions were statistically significant except for the interaction between SNP rs1121923 and fat intake (energy %) on HDL-C (*P* = 0.003) (Table 3), where among those who consumed a high fat diet (3^rd^ tertile: 28.4 *±* 2.5%), the T allele carriers had significantly higher HDL-C concentrations compared to the CC homozygotes (*P* = 0.0002) (Fig. 1a). To test whether this interaction was significant on HDL-C as a categorical variable, we stratified the data based on normal and low HDL-C levels according to the NCEP ATP III guidelines for dyslipidemia [33] and found that among those who consumed a high fat diet, the individuals who carried the T allele had 30% reduced risk of low HDL-C levels compared to the CC homozygotes (*P* = 0.001) (Fig. 1b). We further investigated the interaction of the SNP with various fat subclass intakes on HDL-C but none of the interactions were statistically significant [monounsaturated (MUFA) (*P* = 0.36), polyunsaturated (PUFA) (*P* = 0.22) and saturated fatty acids (SFA) (*P* = 0.46)].

**Table 3:** Interaction between lipoprotein lipase single nucleotide polymorphisms and dietary factors on HDL-C levels

Beta coefficients ± standard error (P_interaction_) for interaction on HDL-C (continuous variable)		
Interaction between rs4922115* fat energy intake (%)	Interaction between rs4922115* protein energy intake (%)	Interaction between rs4922115* carbohydrate energy intake (%)
−0.01 ± 0.002 (0.1)	−0.02 ± 0.01 (0.2)	0.01 ± 0.02 (0.06)
Interaction between rs1121923* fat energy intake (%)	Interaction between rs1121923* protein energy intake (%)	Interaction between rs1121923* carbohydrate energy intake (%)
−0.01 ± 0.01 (**0.003**)	−0.3 ± 0.01 (0.02)	0.01 ± 0.002 (0.05)
Interaction between rs328* fat energy intake (%)	Interaction between rs328* protein energy intake (%)	Interaction between rs328* carbohydrate energy intake (%)
−0.01 ± 0.002 (0.16)	−0.02 ± 0.01 (0.12)	0.01 ± 0.002 (0.07)
Interaction between rs285* fat energy intake (%)	Interaction between rs285* protein energy intake (%)	Interaction between rs285* carbohydrate energy intake (%)
0.01 ± 0.002 (0.05)	0.02 ± 0.01 (0.04)	−0.01 ± 0.002 (0.03)

*HDL-C* High density lipoprotein cholesterol

*P*_interaction_ values adjusted for age, gender, body mass index, type 2 diabetes and total energy intake

(*) refers to the interaction between SNP and dietary factor

Those p values that are in bold implicates those values that are significant after Bonferroni correction

The SNP rs285 alone showed a significant association with serum TAG (*P* = 1 × 10^−5^), where CC genotype carriers had higher TAG concentrations than T allele carriers (Table 4). In the interaction analysis, there was an interaction of the *LPL* SNP rs4922115 with fat intake (energy %) on TAG, where, among those who consumed a low (1^st^ tertile: 18.1 ± 2.6%) or medium fat (2nd tertile: 23.4 ± 1.1%) diet, individuals carrying the GG genotype had significantly lower TAG concentrations compared to ‘A’ allele carriers (*P* = 0.01 for low fat intake; *P* = 0.02 for medium fat intake). However, after correction for multiple testing, this interaction was not statistically significant. None of the other interactions between the SNPs and dietary factors on total cholesterol, serum TAG and LDL-C were statistically significant (Additional file 1: Table S1).

**Table 4:** Association between single nucleotide polymorphisms (SNPs) at lipoprotein lipase gene and lipid traits

SNPs	Total cholesterol mmol/l	Triglycerides mmol/l	LDL-C mmol/l
SNP rs4922115			
GG	4.9 ± 1.1	1.6 ± 1.0	3.1 ± 0.9
GA	4.8 ± 1.1	1.9 ± 1.8	3.0 ± 0.9
AA	4.7 ± 0.9	1.7 ± 0.8	2.9 ± 0.8
Dominant model (GG vs GA + AA) (*P* value)	0.4	0.001	0.1
SNP rs1121923			
CC	4.8 ± 1.1	1.7 ± 1.4	3.0 ± 0.9
CT	5.1 ± 1.0	1.7 ± 1.0	3.2 ± 0.9
TT	4.9 ± 0.6	1.4 ± 0.4	3.3 ± 0.4
Dominant model (CC vs CT + TT) (*P* value)	0.4	0.3	0.9
SNP rs328			
CC	4.9 ± 1.0	1.7 ± 1.1	3.1 ± 0.9
CG	4.9 ± 1.1	1.5 ± 0.9	3.1 ± 0.9
GG	5.0 ± 0.9	1.4 ± 0.5	3.2 ± 0.8
Dominant model (CC vs CG + GG) (*P* value)	0.3	0.1	0.3
SNP rs285			
CC	4.8 ± 1.1	1.7 ± 1.2	3.0 ± 0.8
CT	4.8 ± 1.1	1.5 ± 0.9	3.1 ± 0.9
TT	4.8 ± 1.0	1.4 ± 0.8	3.0 ± 0.8
Dominant model (CC vs CT + TT) (*P* value)	0.7	**0.00009**	0.4

*LDL-c* Low density lipoprotein cholesterol

Results are expressed as mean ± SD

*P* values adjusted for age, gender, body mass index and type 2 diabetes

Those p values that are in bold implicates those values that are significant after Bonferroni correction

## Discussion

To our knowledge, this is the first genetic epidemiological study to investigate the interaction between SNPs at *LPL* gene and dietary factors on blood lipids in an Asian Indian population. Our study provides evidence for a novel interaction between SNP rs1121923 and fat intake (energy %) on HDL-C, where the T allele carriers had significantly higher levels of HDL-C compared to the CC homozygotes among those who consumed a high fat diet. Given that the total fat intake has increased in India in the last few decades and Asian Indians are characterized by altered lipid levels and at a higher risk of premature coronary artery disease (CAD) [38], our study findings have significant public health implications.

Several *LPL* polymorphisms have been extensively studied in association with various lipid traits [12, 22, 25, 26]. The most notable of these known functional common polymorphisms is rs328, also known as S447X (premature truncation at codon 447). SNP rs328 is a gain-of-function polymorphism that has been shown to be consistently associated with higher HDL-C [12]. Our study has also shown a significant association of the SNP rs328 with HDL-C concentrations, where the minor ‘G’ allele carriers had significantly higher HDL-C compared to those with common CC genotype. The rs285 (*Pvu II)* variant located in the intron 6 of the *LPL* gene has been shown to be associated with dyslipidemic phenotypes such as low HDL-C and high TAG among Caucasians [39], which is in accordance with our study findings in Asian Indians where the CC genotype carriers of the SNP rs285 had significantly higher TAG than T allele carriers. The ‘A’ allele of the SNP rs4922115 (located in the 3′ UTR region) and ‘C’ allele of the SNP rs1121923 (Val135Val) (located in the exon 3) were also associated with lower HDL-C levels in our study; however, after correction for multiple testing they were not significant. Our findings confirm the previously reported associations and reveal that *LPL* SNPs play an important role in lipid metabolism in this Asian Indian population.

Total fat intake has increased considerably in India in the last few decades [40]. The National Sample Survey Organization survey has reported that the fat intake of urban component of Indian populations has increased from 42.0 g/d/capita in 1993–1994 to 52.5 g/d/capita in 2011–2012 [41]. Interestingly our study in this South Indian population has identified an interaction between *LPL* SNP rs1121923 and fat intake (energy %) on HDL-C, where, among those who consumed a high fat diet (28.4%), individuals carrying the T allele had significantly higher HDL-C concentrations compared to the CC genotype carriers. Even though our study is the first to report this gene-diet interaction, previous studies in developing countries have shown that the quantity of dietary fats can affect the lipid profile and is directly related to the development of metabolic diseases such as obesity and diabetes [42, 43]. Quality of dietary fat has also shown to alter blood lipid levels [43] but the present study failed to identify an interaction of the *LPL* SNP with MUFA, PUFA and SFA, respectively, on lipid traits. Vegetable oils used in Indian cooking represent 80% of the visible fat consumed [44] and a study in an Indian population showed that the higher ratio of n_6_:n_3_ was attributed to the type and quantity of oil used [45]. Previous studies have shown that dietary fat increases HDL-C [46], which is partially explained by our study findings where the T allele carriers of the SNP rs1121923, who consumed a high fat diet, had higher HDL-C concentrations compared to CC genotype carriers. Even though the exact mechanism by which T allele contributes to the increase in HDL-C levels under conditions of high fat diet is unknown, the finding is suggestive of the complex inheritance pattern of the HDL-C levels [47], where several genes/polymorphisms are likely to contribute to the alteration of HDL-C levels through gene-gene and gene-diet interactions. Our findings are supported by animal studies [48] where mice that are challenged with a high fat diet showed a strong correlation between LPL activity and HDL cholesterol suggesting the link between LPL, fat intake and HDL levels.

Besides HDL-C, there were interactions of the *LPL* SNP rs4922115 with fat energy intake (%) on TAG, where, among those who consumed a low or medium fat diet, individuals carrying the GG genotype had significantly lower TAG concentrations compared to ‘A’ allele carriers. The SNP rs4922115 has not been studied previously in other populations except in Hispanics [49], where this SNP was identified by direct sequencing. However, the study did not explore the individual effect of the SNP on blood lipids and hence we are unable to compare our findings with the Hispanic study. Even though the interaction between SNP rs4922115 and fat energy intake (%) was not statistically significant after Bonferroni correction in our study, replication of these interactions in another large cohort is highly warranted.

One of the main limitations of the study is the small sample size. Given that there are no previously reported effect sizes for the *LPL* SNP-diet interaction on blood lipids, we are unable to calculate the statistical power for our study. However, we were still able to identify significant gene-diet interactions on HDL-C even after correction for multiple testing. The interaction was significant only with total fat intake (energy %) but not when split as PUFA, MUFA and SFA, which might be due to small sample size. Nevertheless, our study is well powered to identify the effect sizes for association between the SNPs and lipids. To increase the statistical power, individuals with and without T2D were included and hence it is possible that the T2D status could have introduced a bias in our study as the dietary pattern is likely to be changed among ‘known’ diabetic participants. However, T2D status was adjusted in all the analyses and the interaction findings were borderline significant even after excluding the ‘known’ diabetic participants from our analysis (data not shown), which could also be due to the small sample size after exclusion. Another limitation is that, our study was cross-sectional and therefore was unable to examine the casual relationship between fat intake and lowering of HDL-C levels; randomized controlled trials with prospective genotyping are required to explore the causality using genetic markers. The main strength of the present study is that a validated interviewer-administered FFQ was used to measure the usual long-term intake of the population. Furthermore, the sampling is representative of the overall population of Chennai. Indeed, the intake of major foods in our study was similar to the findings of the pooled urban data of the National Nutrition Monitoring Bureau for ten states in India [50] and hence, the results of the present study could be reasonably extrapolated to urban Indian population.

## Conclusions

Our study confirms the association between *LPL* SNP rs328 and HDL-C concentrations and also provides an evidence for a novel interaction between SNP rs1121923 and fat intake (energy %) on HDL-C levels in this Asian Indian population. Given that Asian Indians have altered lipid profile and an increased predisposition to premature CAD [38, 40], our study suggesting that individuals carrying T allele of the SNP rs1121923 have increased HDL-C levels when consuming a high fat diet has significant public health implications. This finding warrants confirmation in prospective studies and randomized controlled trials.

## Additional file

Additional file 1: Table S1. Interaction between single nucleotide polymorphisms (SNPs) at lipoprotein lipase gene and dietary factors on lipids traits. (DOCX 17 kb)

## Abbreviations

BMI: Body mass index
CAD: Coronary artery disease
CURES: Chennai urban rural epidemiological study
CVD: Cardiovascular disease
FFQ: Food frequency questionnaire
HDL-C: High-density lipoprotein cholesterol
HWE: Hardy–Weinberg equilibrium
LDL-C: Low density lipoprotein cholesterol
*LPL*: Lipoprotein lipase
MUFA: Monounsaturated
NCEP-ATP III: National Cholesterol Education Program-Adult Treatment Panel III, HbA1c, Glycated haemoglobin
NGT: Normal glucose tolerance
PUFA: Polyunsaturated
SFA: Saturated fatty acids
SNPs: Single nucleotide polymorphisms
T2D: Type 2 diabetes
TAG: Triacylglycerol
