# Supplementary material for: High fat diet modifies the association of lipoprotein lipase gene polymorphism with high density lipoprotein cholesterol in an Asian Indian population
Source: Nutr Metab (Lond). 2017 Jan 19;14:8. doi: 10.1186/s12986-016-0155-1 (PMC5247808; doi:10.1186/s12986-016-0155-1)
Supplement: Additional file 1: Table S1. — Interaction between single nucleotide polymorphisms (SNPs) at lipoprotein lipase gene and dietary factors on lipids traits. (DOCX 17 kb) [file 12986_2016_155_MOESM1_ESM.docx]

Additional file

**Additional file 1: Table S1. Interaction between single nucleotide polymorphisms (SNPs) at lipoprotein lipase gene and dietary factors on lipids traits**

| **Beta coefficients (standard error) P_interaction_* for interaction of SNP rs4922115 with dietary factors on total cholesterol** | | |
| --- | --- | --- |
| *Interaction between rs4922115* fat energy intake* | *Interaction between rs4922115* protein energy intake* | *Interaction between rs4922115* carbohydrate energy intake* |
| -0.1 (0.5)  0.8 | -0.2 (1.9)  0.9 | -0.01 (0.4)  0.9 |
| **Beta coefficients (standard error) P_interaction_* for interaction of SNP rs4922115 with dietary factors on serum triglycerides** | | |
| *Interaction between rs4922115* fat energy intake* | *Interaction between rs4922115* protein energy intake* | *Interaction between rs4922115* carbohydrate energy intake* |
| -3.5 (1.4)  0.01 | 7.4 (5.4)  0.2 | -0.8 (1.1)  0.5 |
| **Beta coefficients (standard error) P_interaction_* for interaction of SNP rs4922115 with dietary factors on LDL-C** | | |
| *Interaction between rs4922115* fat energy intake* | *Interaction between rs4922115* protein energy intake* | *Interaction between rs4922115* carbohydrate energy intake* |
| -0.03 (0.4)  0.9 | 0.61 (1.6)  0.7 | -0.08 (0.3)  0.8 |
| **Beta coefficients (standard error) P_interaction_* for interaction of SNP rs1121923 with dietary factors on total cholesterol** | | |
| *Interaction between rs1121923* fat energy intake* | *Interaction between rs1121923* protein energy intake* | *Interaction between rs1121923* carbohydrate energy intake* |
| -0.2 (0.7)  0.8 | 0.5 (2.4)  0.8 | 0.03 (0.5)  0.9 |
| **Beta coefficients (standard error) P_interaction_* for interaction of SNP rs1121923 with dietary factors on serum triglycerides** | | |
| Interaction between rs1121923* fat energy intake (%) | Interaction between rs1121923* protein energy intake (%) | Interaction between rs1121923* carbohydrate energy intake (%) |
| 2.5 (1.9)  0.2 | 3.8 (6.8)  0.6 | -2.5 (1.5)  0.09 |
| **Beta coefficients (standard error) P_interaction_* for interaction of SNP rs1121923 with dietary factors on LDL-C** | | |
| Interaction between rs1121923* fat energy intake (%) | Interaction between rs1121923* protein energy intake (%) | Interaction between rs1121923* carbohydrate energy intake (%) |
| -0.2 (0.6)  0.7 | 0.9 (1.9)  0.6 | 0.1 (0.4)  0.8 |
| **Beta coefficients (standard error) P_interaction_* for interaction of SNP rs328 with dietary factors on total cholesterol** | | |
| Interaction between rs328* fat energy intake (%) | Interaction between rs328* protein energy intake (%) | Interaction between rs328* carbohydrate energy intake (%) |
| 0.5 (0.5)  0.3 | 0.1 (0.2)  0.7 | -0.1 (0.4)  0.8 |
| **Beta coefficients (standard error) P_interaction_* for interaction of SNP rs328 with dietary factors on serum triglycerides** | | |
| Interaction between rs328* fat energy intake (%) | Interaction between rs328* protein energy intake (%) | Interaction between rs328* carbohydrate energy intake (%) |
| 0.6 (1.3)  0.6 | -0.6 (5.0)  0.9 | -0.7 (0.9)  0.5 |
| **Beta coefficients (standard error) P_interaction_* for interaction of SNP rs328 with dietary factors on LDL-C** | | |
| Interaction between rs328* fat energy intake (%) | Interaction between rs328* protein energy intake (%) | Interaction between rs328* carbohydrate energy intake (%) |
| 0.7 (0.5)  0.1 | -0.2 (0.2)  0.3 | -0.4 (0.3)  0.3 |
| **Beta coefficients (standard error) P_interaction_* for interaction of SNP rs285 with dietary factors on total cholesterol** | | |
| Interaction between rs285* fat energy intake (%) | Interaction between rs285* protein energy intake (%) | Interaction between rs285* carbohydrate energy intake (%) |
| -0.2 (0.4)  0.5 | -0.8 (1.5)  0.6 | 0.3 (0.3)  0.3 |
| **Beta coefficients (standard error) P_interaction_* for interaction of SNP rs285 with dietary factors on serum triglycerides** | | |
| Interaction between rs285* fat energy intake | Interaction between rs285* protein energy intake | Interaction between rs285* carbohydrate energy intake |
| -1.6 (0.9)  0.1 | -1.0 (3.4)  0.8 | 1.2 (0.7)  0.08 |
| **Beta coefficients (standard error) P_interaction_* for interaction of SNP rs285 with dietary factors on LDL-C** | | |
| Interaction between rs285* fat energy intake | Interaction between rs285* protein energy intake | Interaction between rs285* carbohydrate energy intake |
| -0.09 (0.3)  0.8 | -1.0 (1.3)  0.5 | 0.1 (0.3)  0.7 |

P_interaction_ values adjusted for age, gender, body mass index, type 2 diabetes and total energy intake
